# Autosomal recessive agammaglobulinemia due to compound heterozygous IGHM alterations identified: a case report

**DOI:** 10.3389/fmed.2026.1901229

**Published:** 2026-08-19

**Authors:** Cristhel Puente, Juan Carlos Aldave Becerra

**Affiliations:** Edgardo Rebagliati Martins Hospital, Lima, Peru

**Keywords:** agammaglobulinemia, exome sequencing, immunodeficiency, inborn error immunity, recessive hereditary diseases

## Abstract

**Background:**

Agammaglobulinemia type 1 (AGM1) is a rare autosomal recessive inborn error of immunity caused by biallelic defects in the immunoglobulin heavy constant mu gene (IGHM), resulting in arrested B-cell development, profound antibody deficiency, and recurrent bacterial infections. Owing to its rarity and genetic heterogeneity, establishing a molecular diagnosis may remain challenging despite extensive genetic evaluation.

**Case presentation:**

An 11-year-old girl presented with recurrent respiratory infections, profound hypogammaglobulinemia, and absent circulating B cells since infancy. Intravenous immunoglobulin replacement therapy was initiated early in life, resulting in marked clinical improvement. Previous targeted next-generation sequencing using a 407-gene panel for inborn errors of immunity did not establish a molecular diagnosis. Because of the persistent clinical suspicion of a monogenic disorder, trio whole-exome sequencing combined with copy number variant analysis was subsequently performed. This identified compound heterozygous alterations in IGHM, consisting of a paternally inherited missense variant (c.1763C>G; p.His417Asp) and a maternally inherited 169.1-kb deletion encompassing the entire IGHM gene. Segregation analysis confirmed that both alterations were located in trans. Although both variants remain classified as variants of uncertain significance according to American College of Medical Genetics and Genomics (ACMG) criteria, the characteristic clinical phenotype, longitudinal immunological findings, segregation pattern, and biological plausibility strongly support their clinical relevance.

**Conclusion:**

This case expands the molecular spectrum of IGHM-associated agammaglobulinemia and highlights the diagnostic value of integrating whole-exome sequencing with copy number variant analysis in patients with unresolved primary antibody deficiencies, particularly when targeted genetic testing is inconclusive.

## 1 Introduction

Agammaglobulinemia comprises a heterogeneous group of rare inherited disorders characterized by profoundly reduced or absent serum immunoglobulins resulting from defects in early B-cell development. Affected individuals typically present during infancy or early childhood with recurrent bacterial infections, markedly impaired antibody production, and absent or severely reduced circulating B lymphocytes. Early diagnosis and prompt initiation of immunoglobulin replacement therapy are essential to prevent irreversible organ damage and improve long-term outcomes. Although pathogenic variants in the Bruton tyrosine kinase (*BTK*) gene account for approximately 85% of cases of agammaglobulinemia, the remaining cases result from defects in other genes involved in pre-B-cell receptor formation and signaling, giving rise to genetically heterogeneous autosomal recessive forms of the disease (1, 2).

Among these disorders, agammaglobulinemia type 1 (AGM1; OMIM #601495) is caused by biallelic pathogenic variants affecting the immunoglobulin heavy constant mu (*IGHM*) gene, which encodes the *μ* heavy chain of immunoglobulin M. The μ heavy chain is an essential structural component of the pre-B-cell receptor (pre-BCR), a critical checkpoint required for the transition from the pro-B-cell to the pre-B-cell stage during normal B-cell development. Disruption of this pathway results in early developmental arrest of the B-cell lineage, leading to profound hypogammaglobulinemia and an almost complete absence of mature peripheral B cells (3).

To date, only a small number of patients with *IGHM*-associated agammaglobulinemia have been reported, reflecting the exceptional rarity of this disorder (3–5). Most affected patients develop recurrent sinopulmonary infections during the first years of life and exhibit a characteristic immunological phenotype consisting of absent or markedly reduced circulating B cells, severe hypogammaglobulinemia, and preserved T-cell immunity (3–5). Reported pathogenic mechanisms include nonsense, splice-site, and missense variants, as well as large deletions involving the *IGHM* locus, either homozygous or compound heterozygous (3–5). Despite these characteristic clinical features, establishing a molecular diagnosis remains challenging because complex genetic mechanisms, particularly copy number variants (CNVs), may be missed by sequencing-only approaches (5).

Recent advances in next-generation sequencing have substantially improved the molecular diagnosis of inborn errors of immunity. However, whole-exome sequencing (WES) alone may not identify structural genomic alterations unless dedicated CNV analysis is performed. Therefore, integrating sequence and structural variant analysis has become increasingly important in patients with unresolved primary antibody deficiencies, particularly when targeted gene panels fail to identify the underlying genetic defect (6).

Here, we report the case of an 11-year-old girl with a longstanding diagnosis of agammaglobulinemia in whom previous targeted next-generation sequencing using a 407-gene panel for inborn errors of immunity failed to establish a molecular diagnosis. Comprehensive genomic evaluation using trio whole-exome sequencing (WES) combined with copy number variant (CNV) analysis identified compound heterozygous alterations in *IGHM*, consisting of a paternally inherited missense variant and a maternally inherited whole-gene deletion. Although both variants remain classified as variants of uncertain significance (VUS) according to the American College of Medical Genetics and Genomics (ACMG) criteria, the integration of the patient’s clinical presentation, immunological phenotype, segregation analysis, and genomic findings strongly supports their clinical relevance. This case expands the molecular spectrum of *IGHM*-associated agammaglobulinemia and highlights the importance of combining complementary genomic approaches to improve the molecular diagnosis of rare primary antibody deficiencies.

## 2 Case description

An 11-year-old girl was referred to our Immunology Department for continued follow-up of a primary antibody deficiency characterized by profound hypogammaglobulinemia and absent circulating B lymphocytes. She was born at 37 weeks of gestation by cesarean section because of maternal preeclampsia, with a birth weight of 3,176 g. The perinatal period was uneventful, with no history of significant neonatal infections or other complications.

During infancy, she experienced recurrent episodes of otitis media, bronchiolitis, and upper respiratory tract infections requiring multiple courses of antibiotic therapy. At 1 year and 11 months of age, she was hospitalized because of progressive unilateral ptosis associated with generalized weakness. Immunological evaluation performed during hospitalization revealed profound hypogammaglobulinemia and an almost complete absence of circulating B cells, leading to the diagnosis of agammaglobulinemia. Intravenous immunoglobulin (IVIG) replacement therapy was initiated shortly thereafter, at approximately 2 years of age, and has been maintained regularly since diagnosis.

Despite regular IVIG replacement therapy, the patient continued to experience intermittent respiratory manifestations during childhood, including recurrent upper respiratory tract infections and chronic productive cough, although both the frequency and severity of infections progressively decreased after treatment initiation. She also developed recurrent gastrointestinal symptoms characterized by abdominal pain, nausea, vomiting, and intermittent diarrhea.

In addition to her infectious phenotype, several non-immunological manifestations were identified during follow-up. These included amelogenesis imperfecta with dentin defects, persistent unilateral ptosis, generalized joint hypermobility, delayed bone age, and short stature. Owing to the complexity of her clinical presentation, she underwent multidisciplinary evaluation by specialists in Immunology, Pulmonology, Gastroenterology, Neurology, Endocrinology, Genetics, and Dentistry.

Family history was unremarkable, with no known history of primary immunodeficiency, recurrent severe infections, early childhood deaths, consanguinity, or other hereditary disorders.

Physical examination at the most recent evaluation demonstrated proportionate short stature, persistent unilateral ptosis, generalized joint hypermobility, and dental abnormalities consistent with amelogenesis imperfecta. No clinically significant lymphadenopathy or hepatosplenomegaly was observed.

The patient has remained on regular intravenous immunoglobulin (IVIG) replacement therapy, administered every 21–28 days, and is not receiving any additional immunomodulatory treatment. She is currently treated with inhaled fluticasone/salmeterol (125 μg once daily) for asthma and intranasal budesonide every other day for allergic rhinitis.

Long-term IVIG replacement therapy has resulted in a marked reduction in severe infections and hospitalizations, leading to progressive clinical stabilization and improved quality of life. Pulmonary surveillance included a non-contrast chest computed tomography (CT) performed in May 2025, which demonstrated only a small linear atelectatic band in the right middle lobe, without evidence of bronchiectasis, pulmonary nodules, active infectious or inflammatory changes, pleural or pericardial effusion, or significant mediastinal lymphadenopathy. A 6-min walk test demonstrated normal exercise tolerance, with a walking distance of 554 m (96.7% of the predicted value), without oxygen desaturation or exercise limitation. Spirometry, performed in June 2025, showed normal pulmonary function with a significant bronchodilator response in FEV₁.

At the most recent follow-up, the patient remained clinically stable, with no evidence of bronchiectasis or other chronic structural lung disease. These findings are consistent with the favorable clinical course observed after long-term IVIG replacement therapy. Treatment adherence has been excellent, and no significant adverse events related to IVIG have been reported.

The chronological evolution of the patient’s clinical course, immunological findings, genetic investigations, and therapeutic interventions is summarized in Table 1.

**Table 1:** Timeline of the patient’s clinical course.

Age/year	Clinical events	Diagnostic evaluation	Treatment/Outcome
Birth (37 weeks)	Born by cesarean section due to maternal preeclampsia; uneventful neonatal period	—	—
Infancy	Recurrent otitis media, bronchiolitis, and upper respiratory tract infections	Initial clinical evaluation	Multiple courses of antibiotics
1 year 11 months	Hospitalization due to progressive unilateral ptosis and generalized weakness	Severe hypogammaglobulinemia and absent circulating B cells identified	Diagnosis of agammaglobulinemia established
2 years	Persistent antibody deficiency	Immunological evaluation confirmed profound humoral immunodeficiency	Intravenous immunoglobulin (IVIG) replacement initiated
Childhood	Recurrent respiratory infections, chronic productive cough, abdominal pain, nausea, vomiting, intermittent diarrhea	Multidisciplinary evaluation (Pulmonology, Gastroenterology, Neurology, Endocrinology, Genetics, Dentistry)	Continued IVIG replacement therapy
2025	Persistent respiratory symptoms under follow-up	Chest CT: small linear atelectatic band in the right middle lobe; no bronchiectasis or active pulmonary disease. Six-minute walk test normal (554 m, 96.7% predicted). Spirometry normal with bronchodilator response	Continued IVIG, inhaled fluticasone/salmeterol, intranasal budesonide
2025	Previous targeted panel inconclusive	407-gene panel for IEI did not identify the molecular diagnosis	Proceeded to trio WES + CNV analysis
2025	Molecular diagnosis established	Trio WES + CNV identified compound heterozygous *IGHM* alterations (c.1763C > G and whole-gene deletion)	Diagnosis of IGHM-associated agammaglobulinemia supported

## 3 Diagnostic assessment

Serial immunological evaluations consistently demonstrated a profound defect in humoral immunity. Initial lymphocyte subset analysis performed in 2017 revealed a complete absence of circulating B cells, while T-cell populations were preserved (CD3 + 2,300 cells/μL, CD4 + 1,001 cells/μL, CD8 + 1,276 cells/μL), with normal NK-cell counts (526 cells/μL).

In 2018, while receiving regular intravenous immunoglobulin (IVIG) replacement therapy, serum immunoglobulin levels showed an IgG concentration of 534 mg/dL, undetectable IgA, and an IgM level of 1 mg/dL. Flow cytometry demonstrated preserved T-cell populations (CD3 + 3,276 cells/μL, CD4 + 2,219 cells/μL, CD8 + 864 cells/μL), markedly reduced B cells (CD19 + 301 cells/μL), and decreased NK-cell counts (71 cells/μL).

Subsequent evaluations confirmed persistent impairment of humoral immunity. In 2021, serum immunoglobulin levels remained profoundly abnormal despite IVIG replacement (IgA 0 mg/dL, IgG 1,092 mg/dL, IgM 0 mg/dL). Lymphocyte subset analysis demonstrated persistent B-cell lymphopenia (CD19 + 42 cells/μL), whereas T-cell populations remained preserved (CD3 + 2,181 cells/μL, CD4 + 1,366 cells/μL, CD8 + 586 cells/μL), with NK-cell counts of 353 cells/μL.

The most recent immunological evaluation, performed in 2025, again demonstrated an absence of circulating B cells, while T-cell and NK-cell populations remained preserved (CD3 + 2,427 cells/μL, CD4 + 1,473 cells/μL, CD8 + 636 cells/μL, NK cells 144 cells/μL). Serum immunoglobulin concentrations continued to demonstrate profound antibody deficiency despite ongoing replacement therapy (IgA < 2 mg/dL, IgG 873 mg/dL, IgM < 10 mg/dL).

Overall, the longitudinal immunological profile strongly suggested a severe defect affecting early B-cell development and was highly consistent with congenital agammaglobulinemia. The evolution of serum immunoglobulin concentrations and lymphocyte subsets is summarized in Table 2.

**Table 2:** Longitudinal immunological evaluation.

Year	Age	IgG (mg/dL)	IgA (mg/dL)	IgM (mg/dL)	CD19 + (cells/μL)	CD3+	CD4+	CD8+	NK cells
2017	2 years	NA	NA	NA	**0**	2,300	1,001	1,276	526
2018*	3 years	534	0	1	301	3,276	2,219	864	71
2021*	6 years	1,092	0	0	42	2,181	1,366	586	353
2025*	11 years	873	<2	<10	**0**	2,427	1,473	636	144

*Measurements obtained while receiving regular intravenous immunoglobulin replacement therapy.

Despite the highly characteristic clinical and immunological phenotype, previous targeted next-generation sequencing using a 407-gene panel for inborn errors of immunity identified several heterozygous variants of uncertain significance, including variants in *CARMIL2, DUOX2, IL17RC, MYO5B, NBAS, PARN, PRKDC, SLC46A1, TFRC,* and *TLR3*, but failed to establish a definitive molecular diagnosis. Given the persistent suspicion of a monogenic disorder affecting early B-cell development, comprehensive genomic evaluation was subsequently performed using trio whole-exome sequencing (WES) in the proband and both parents, combined with copy number variant (CNV) analysis.

Given the persistent clinical suspicion of a monogenic disorder despite previous targeted genetic testing, the patient underwent trio whole-exome sequencing (WES) with copy number variant (CNV) analysis through a certified diagnostic laboratory. The analysis identified a heterozygous missense variant in *IGHM* and a heterozygous deletion encompassing the entire *IGHM* gene. Segregation analysis demonstrated paternal inheritance of the missense variant and maternal inheritance of the deletion, confirming that both alterations were located *in trans* and establishing a compound heterozygous genotype. Variant classification was performed according to the American College of Medical Genetics and Genomics (ACMG) guidelines.

Trio WES identified a heterozygous IGHM missense variant, c.1763C > G (GRCh38: chr14:105,854,456G > C), inherited from the father. In transcript ENST00000637539, this variant is predicted to result in a p.His417Asp amino acid substitution. The affected residue lies within a functionally important region of the *μ* heavy chain, where pathogenic variants involving nearby amino acids, including p.Cys413Gly and p.Glu434Lys, have previously been associated with agammaglobulinemia.

CNV analysis identified a maternally inherited heterozygous 169.1-kb interstitial deletion at chromosome 14q32.33 [seq(GRCh38) 14q32.33(105,731,567_105,900,654)x1], encompassing the entire IGHM gene. Segregation analysis demonstrated paternal inheritance of the missense variant and maternal inheritance of the deletion, confirming that both alterations were located in trans, establishing a compound heterozygous genotype. No additional orthogonal confirmation using MLPA, quantitative PCR, or chromosomal microarray was performed.

Both the missense variant and the whole-gene deletion remain classified as variants of uncertain significance (VUS) according to ACMG criteria. Nevertheless, several lines of evidence support their clinical relevance, including: (i) a highly specific clinical phenotype of congenital agammaglobulinemia, (ii) persistent profound hypogammaglobulinemia with absent circulating B cells, (iii) segregation of both variants in trans, and (iv) the presence of previously reported pathogenic variants affecting the same functional region of the *μ* heavy chain. Collectively, these findings strongly support the biological relevance of the identified genotype; however, functional studies are required before definitive pathogenicity can be established.

Based on the integration of the clinical presentation, immunological phenotype, segregation analysis, and genomic findings, the patient’s phenotype was considered highly consistent with IGHM-associated agammaglobulinemia (AGM1).

## 4 Discussion

Agammaglobulinemia type 1 (AGM1) is a rare autosomal recessive inborn error of immunity caused by biallelic defects affecting the *IGHM* gene, which encodes the *μ* heavy chain of immunoglobulin M. The μ heavy chain is an essential component of the pre-B-cell receptor (pre-BCR), a critical checkpoint required for the transition from the pro-B-cell to the pre-B-cell stage during normal B-cell development. Failure to assemble a functional pre-BCR results in an early developmental arrest of the B-cell lineage, leading to profound hypogammaglobulinemia, absent or markedly reduced circulating B cells, and recurrent bacterial infections beginning in early childhood (1, 3).

The clinical and immunological findings in our patient are highly consistent with the phenotype previously described in *IGHM*-associated agammaglobulinemia. Since infancy, she experienced recurrent respiratory infections and persistent antibody deficiency characterized by absent or profoundly reduced circulating B cells, while T-cell and NK-cell populations remained preserved throughout serial evaluations. This immunological profile is characteristic of disorders affecting early B-cell development and supports a defect at the pre-B-cell stage, as previously demonstrated in patients with *IGHM* deficiency (3, 5).

Only a limited number of patients with *IGHM*-associated agammaglobulinemia have been reported in the literature. The first molecularly characterized cases demonstrated that large deletions involving the *IGHM* locus or biallelic pathogenic variants disrupting *μ* heavy-chain expression result in developmental arrest between the pro-B-cell and pre-B-cell stages, leading to absent mature B cells and profound antibody deficiency (3). Subsequently, additional reports expanded the mutational spectrum to include nonsense, splice-site, missense, and compound heterozygous variants, further establishing *IGHM* deficiency as a distinct cause of autosomal recessive agammaglobulinemia (4, 5).

The present case shares several clinical features with previously reported patients, including recurrent respiratory infections beginning during infancy, profound hypogammaglobulinemia, absent or markedly reduced circulating B cells, preserved T-cell immunity, and a favorable clinical response to immunoglobulin replacement therapy. However, our patient also presented with additional manifestations, including amelogenesis imperfecta, persistent unilateral ptosis, generalized joint hypermobility, delayed bone age, and short stature. Although these features are not classically associated with *IGHM* deficiency, they prompted an extensive multidisciplinary evaluation and contributed to the prolonged diagnostic workup.

An important aspect of this report is the identification of compound heterozygous alterations involving *IGHM* through the integration of trio whole-exome sequencing (WES) and copy number variant (CNV) analysis. Previous targeted next-generation sequencing using a 407-gene panel for inborn errors of immunity failed to establish a molecular diagnosis despite the patient’s highly characteristic immunological phenotype. The subsequent identification of a paternally inherited missense variant (c.1763C > G; p.His417Asp) together with a maternally inherited 169.1-kb deletion encompassing the entire *IGHM* gene illustrates the incremental diagnostic value of combining sequence and structural variant analysis in patients with unresolved primary antibody deficiencies.

Our findings closely resemble those reported by Silva et al., who described a patient carrying a compound heterozygous genotype consisting of a nonsense *IGHM* variant (p.Tyr43*) and a deletion involving the second allele (4). Similarly, Lopez-Granados et al. reported a patient with a point mutation in one allele and a large deletion affecting the second allele, demonstrating that compound heterozygosity involving sequence and structural variants represents an important disease mechanism in *IGHM*-associated agammaglobulinemia (3). More recently, a novel homozygous missense variant (p.Trp259Arg) was reported in a child with absent B cells and profound hypogammaglobulinemia, further expanding the spectrum of clinically relevant *IGHM* missense variants (5). A comparison between previously reported cases and the present patient is summarized in Table 3.

**Table 3:** Comparison of previously reported IGHM-associated agammaglobulinemia cases with the present case.

Study	Patient	Main clinical features	Immunological phenotype	Genetic findings	Outcome
Lopez-Granados et al., 2002 (4)	35-month-old girl	Failure to thrive, chronic diarrhea, recurrent respiratory infections	Profound hypogammaglobulinemia, absent peripheral B cells	Large deletion involving the *IGH* locus, including *IGHM*	Improved after IVIG replacement therapy
Silva et al., 2017 (1)	7-month-old girl	Sepsis, peritonitis, recurrent bacterial infections	Undetectable IgA and IgM, very low IgG, absent CD19 + B cells, developmental arrest at the pro-B to pre-B-cell stage	Compound heterozygous *IGHM* defect: nonsense variant (p. Tyr43*) and whole-gene deletion	Clinical stabilization with IVIG replacement therapy
Joy Heifetz et al., 2024 (6)	5-year-old girl	Pneumonia complicated by empyema, *Haemophilus influenzae* bacteremia, chronic cough, diarrhea, weight loss	Absent B cells, profound hypogammaglobulinemia, absent vaccine-specific antibodies	Homozygous missense variant *IGHM* c.775 T > C (p. Trp259Arg), classified as VUS	Marked improvement following monthly IVIG replacement therapy
Present case	11-year-old girl	Recurrent respiratory infections, chronic productive cough, gastrointestinal symptoms, amelogenesis imperfecta, unilateral ptosis, generalized joint hypermobility, delayed bone age, short stature	Profound hypogammaglobulinemia, absent or markedly reduced circulating B cells, preserved T-cell immunity	Compound heterozygous *IGHM* alterations: paternal missense variant c.1763C > G (p. His417Asp) and maternally inherited 169.1-kb whole-gene deletion	Long-term clinical stability with IVIG replacement therapy; no evidence of bronchiectasis or chronic structural lung disease

The missense variant identified in our patient (c.1763C > G; p.His417Asp) remains classified as a variant of uncertain significance (VUS) according to ACMG criteria. Nevertheless, several observations support its potential biological relevance. First, the variant affects a highly conserved region of the *μ* heavy chain. Second, pathogenic variants involving nearby amino acid residues, including p.Cys413Gly and p.Glu434Lys, have previously been associated with agammaglobulinemia (3). Third, segregation analysis demonstrated that the missense variant is located in trans with a whole-gene deletion inherited from the opposite parent. Finally, the patient’s phenotype is highly specific for an early B-cell developmental defect, providing strong genotype–phenotype correlation. Although these findings do not permit definitive reclassification of the variant, they substantially strengthen the evidence supporting its clinical relevance. Functional studies evaluating *μ* heavy-chain expression or pre-B-cell receptor signaling will be necessary to determine its pathogenicity.

Another important finding is the identification of a whole-gene deletion involving *IGHM*. Large deletions affecting the *IGHM* locus have been reported repeatedly and appear to represent a relatively frequent molecular mechanism in this disorder (3, 4, 6). Such alterations may remain undetected if only sequence variants are analyzed. In our patient, dedicated CNV analysis performed on trio WES data was essential to identify the maternally inherited deletion and establish compound heterozygosity. This case therefore reinforces the importance of incorporating CNV analysis into the routine molecular evaluation of patients with unresolved primary antibody deficiencies, particularly when the clinical phenotype strongly suggests a monogenic disorder but conventional sequencing approaches are inconclusive.

The patient demonstrated a favorable long-term response to IVIG replacement therapy, with a marked reduction in severe infections and hospitalizations. At the most recent follow-up, chest computed tomography showed no evidence of bronchiectasis or other chronic structural lung disease, while pulmonary function testing remained within normal limits. These findings are consistent with the favorable clinical course observed after long-term IVIG replacement therapy and emphasize the importance of early diagnosis and adequate immunoglobulin replacement to preserve long-term pulmonary function (2, 5, 6).

This report has several limitations. Functional studies evaluating the effect of the p.His417Asp substitution on *μ* heavy-chain expression, protein stability, or pre-B-cell receptor signaling were not available. Likewise, protein expression studies were not performed, and the whole-gene deletion identified by CNV analysis was not confirmed using an orthogonal method such as multiplex ligation-dependent probe amplification (MLPA), quantitative PCR, or chromosomal microarray. These additional investigations would further strengthen the molecular evidence and should be considered in future studies.

Despite these limitations, this report has several strengths. It provides comprehensive longitudinal clinical and immunological follow-up over several years, demonstrates segregation of the identified variants within the family, and illustrates the incremental diagnostic value of integrating trio whole-exome sequencing with CNV analysis in patients with unresolved primary antibody deficiencies. Furthermore, this case expands the molecular spectrum of *IGHM*-associated agammaglobulinemia and contributes additional evidence supporting the interpretation of rare *IGHM* variants within the context of detailed clinical, immunological, and segregation data.

## 5 Conclusion

This case describes an 11-year-old girl with a longstanding history of recurrent infections, profound hypogammaglobulinemia, and absent circulating B cells in whom the underlying molecular diagnosis remained unresolved despite previous targeted genetic testing. The integration of trio whole-exome sequencing (WES) and copy-number variant (CNV) analysis identified compound heterozygous alterations in *IGHM*, consisting of a paternally inherited missense variant and a maternally inherited whole-gene deletion.

Although both variants remain classified as variants of uncertain significance (VUS) according to American College of Medical Genetics and Genomics (ACMG) criteria, the patient’s highly characteristic clinical phenotype, longitudinal immunological findings, segregation analysis demonstrating that both variants are located in trans, and the biological relevance of the affected functional region collectively provide strong evidence supporting their clinical significance. Nevertheless, functional studies are required to establish definitive pathogenicity and determine whether future variant reclassification is warranted.

This report expands the molecular spectrum of IGHM-associated agammaglobulinemia and highlights the incremental diagnostic value of integrating trio whole-exome sequencing with CNV analysis in patients with unresolved primary antibody deficiencies, particularly when conventional targeted gene panels fail to identify the underlying genetic defect. Our findings reinforce the importance of interpreting genomic data within the context of detailed clinical and immunological phenotyping and support the use of comprehensive genomic approaches to improve the molecular diagnosis of rare inborn errors of immunity.

## Data Availability

The original contributions presented in this study are included in the article. Additional data are not publicly available because they contain information that could compromise the privacy of the participant. Requests for further information may be directed to the corresponding author.
